# Walkability and socio-economic status in relation to walking, playing and sports practice in a representative Spanish sample of youth: The PASOS study

**DOI:** 10.1371/journal.pone.0296816

**Published:** 2024-03-15

**Authors:** Susana Aznar, Fabio Jimenez-Zazo, Cristina Romero-Blanco, Santiago F. Gómez, Clara Homs, Julia Wärnberg, Maria Medrano, Narcís Gusi, Marcela Gonzalez-Gross, Elena Marín-Cascales, Miguel Ángel González-Valeiro, Lluis Serra-Majem, Nicolás Terrados, Josep A. Tur, Marta Segu, Camille Lassale, Antoni Colom-Fernández, Idoia Labayen, Jesús Sánchez-Gómez, Pedro Emilio Alcaraz, Marta Sevilla-Sanchez, Augusto G. Zapico, Estefanía Herrera-Ramos, Susana Pulgar, Maria del Mar Bibilonii, Clara Sistac, Helmut Schröder, Javier Molina-García

**Affiliations:** 1 PAFS Research Group, Department of Physical Activity and Sports Sciences, Faculty of Sports Sciences, University of Castilla-La Mancha, Toledo, Spain; 2 CIBER of Frailty and Healthy Aging (CIBERFES), Madrid, Spain; 3 PAFS Research Group, Department of Nursery, Physiotherapy and Occupational Therapy, University of Castilla-La Mancha, Ciudad Real, Spain; 4 Gasol Foundation, Sant Boi de Llobregat, Barcelona, Spain; 5 GREpS, Health Education Research Group, Nursing and Physiotherapy Department, University of Lleida, Lleida, Spain; 6 Ciber Epidemiology and Public Health (CIBERESP), Instituto de Salud Carlos III, Spain; 7 Hospital del Mar Medical Research Institute (IMIM), Barcelona, Spain; 8 Cardiovascular Risk and Nutrition Research Group, IMIM (Hospital del Mar Medical Research Institute), Barcelona, Spain; 9 GRoW, Global Research on Wellbeing, Blanquerna School of Life Sciences, University Ramon Llull, Barcelona, Spain; 10 EpiPHAAN Research Group, School of Health Sciences, University of Málaga–Instituto de Investigación Biomédica en Málaga (IBIMA), Málaga, Spain; 11 Centro de Investigación Biomédica en Red Fisiopatología de la Obesidad y la Nutrición (CIBERobn), Institute of Health Carlos III, Madrid, Spain; 12 ELIKOS Group, Institute for Innovation & Sustainable Development in Food Chain (IS-FOOD), Public University of Navarra, Navarra, Spain; 13 Physical Activity and Quality of Life Research Group (AFYCAV), Faculty of Sport Sciences, University of Extremadura, Cáceres, Spain; 14 ImFINE Research Group, Department of Health and Human Performance, Universidad Politécnica de Madrid, Madrid, Spain; 15 Research Center for High Performance Sport, Catholic University of Murcia, Murcia, Spain; 16 Faculty of Sport Sciences, UCAM, Catholic University of Murcia, Murcia, Spain; 17 Faculty of Sports Sciences and Physical Education, University of A Coruña, A Coruña, Spain; 18 Research Institute of Biomedical and Health Sciences (IUIBS), University of Las Palmas de Gran Canaria, Las Palmas, Spain; 19 Preventive Medicine Service, Centro Hospitalario Universitario Insular Materno Infantil (CHUIMI), Canarian Health Service, Las Palmas, Spain; 20 Regional Unit of Sports Medicine, Municipal Sports Foundation of Avilés, Asturias, Spain; 21 Research Group of Community Nutrition & Oxidative Stress, University of the Balearic Islands-IUNICS & Health Research Institute of the Balearic Islands (IDISBA), Palma de Mallorca, Spain; 22 Fundació FC Barcelona, Barcelona, Spain; 23 Department of Language, Arts and Physical Education, Universidad Complutense de Madrid, Madrid, Spain; 24 AFIPS Research Group, Department of Teaching of Physical Education, Arts and Music, University of Valencia, Valencia, Spain; 25 Epidemiology and Environmental Health Joint Research Unit, FISABIO-Universitat Jaume I-Universitat de València, València, Spain; University of Study of Bari Aldo Moro, ITALY

## Abstract

**Purpose:**

Physical activity (PA) provides multiple health-related benefits in children and adolescents, however, at present, the majority of young people are insufficiently physically active. The aim of this study was to evaluate if neighborhood walkability and/or socio-economic status (SES) could affect the practice of walking, play outdoors and sports practice in a representative sample of Spanish children and adolescents.

**Methods:**

A sample of 4092 youth (aged 8–16 years old) from 245 primary and secondary schools in 121 localities from each of the 17 Spanish autonomous communities participated in the study. Walk Score was used to evaluate walkability of the neighborhood and household income was used as an indicator of SES. A 7-item self-reported validated questionnaire, was used to assess PA levels, and in a subsample of 10% of the participants, randomly selected from the entire sample, PA was objectively measured by accelerometers.

**Results:**

Youth from more walkable areas reported more minutes walking per day compared with those from less walkable neighborhoods (51.4 vs 48.8 minutes, respectively). The lowest average minutes spent in playing outdoors was found among participants from low-SES and low-walkable neighborhoods. Neighborhood SES influenced on the participation in team sports during the weekend, being this participation higher in high SES neighborhoods.

**Conclusion:**

Providing high walkable environments seems a good strategy to promote PA regardless SES levels. It seems that improving the walkability is a key component to partially overcome the SES inequalities, especially in urban areas with low SES. High-SES environments can offer better sports facilities and more organized physical activities than low-SES ones.

## Introduction

Physical activity (PA) provides multiple health-related benefits in children and adolescents [1]. Guidelines have been postulated to establish the amount of PA children need to perform to keep healthy [2]. However, 81% of adolescents aged 11–17 years are insufficiently physically active globally, with significant differences in the prevalence of insufficient PA across genders, regions, and countries [3]. In the Spanish context 63.3% of the population aged 8 to 16 does not meet the PA guidelines, i.e. achievement of at least 60 minutes of moderate to vigorous physical activity (MVPA) per day, seven days a week [4].

From an ecological perspective, personal, psychosocial, environmental factors, and policies influence PA behavior [5]. In particular, the GAPPA (Global action plan for physical activity) from the World Health Organization (WHO) explains the four targets’ actions to increase PA levels: make people active, make active societies, promote active environments and active policies [6]. The main message from GAPPA is that behavior needs to be understood within its context. Hence, the let’s be active campaign from GAPPA, has emphasized to promote PA for everyone, everywhere and every day.

On this regard, the association between built environment attributes and PA among youth is growing [7, 8]. The findings from different reviews indicate the existence of a limited evidence and non-conclusive associations between built environment attributes and PA among youth [7, 9]. According to the literature, one of the most supported correlates for PA behavior, especially among the adult population, is neighborhood walkability [7, 10]. Walkability is a concept developed to explain how friendly a neighborhood is to physically active lifestyles [11]. Globally, walkability of an area is usually assessed considering built environmental attributes such as street connectivity, residential density, and land use mix [12].

Associations between walkability and PA are more consistent when studies are based on objective neighborhood evaluation methods, such as geographical information system (GIS) or observational tools, compared to subjective methods (perception instruments) [13]. Nowadays, there is an objectively and publicly available walkability index that is called Walk Score. This indicator is being increasingly used due to its accessibility and comparability in the study of walkability [14]. The limited literature that has analyzed the relationship between Walk Score and PA behavior has been carried out in the adult population and using subjective measures of PA [14]. According to the review by Hall and Ram [14], the validity of Walk Score can vary depending on the type of PA analyzed or socio-demographic characteristics (e.g., age or socio-economic status). The paucity of research in adult population [15–17] suggests the evidence of a positive relationship between Walk Score and the different measures of PA, such as: steps per day, minutes of PA at different intensities or active transportation by walking. Moreover, there is scarce evidence on the relationship between this walkability index and PA among children and adolescents. This current study will attempt to address this lack of information using both subjective and objective measures of PA in children and adolescents.

There are also socioeconomic differences in built environments, suggesting that low-income residents may be exposed to less conducive environments to be active [12, 18]. Moreover, economic resources may exert decisive influences on children’s and adolescents’ PA practice, with those from more advantaged familial background often having higher levels of PA than those in poorer families [19, 20]. Both cultural and material familial resources are important predictors of PA: when parents provide support, their children are more likely to engage in PA [21, 22].

It is therefore unclear whether the association of socioeconomic status (SES) with children’s and adolescents’ practice of PA persists, when other factors, such as environment walkability, are considered. Likewise, there is a lack of studies that analyze these associations in young people using representative samples and carried out in multiple cities or countries [12, 23]. Therefore, the aim of this study was to evaluate if walkability and/or environment socio-economic status could affect the practice of walking, play outdoors and sports practice in a representative sample of Spanish children and adolescents. The representativeness of the study sample is of particular importance to obtain an overall picture of the reality in Spain.

## Materials and methods

### Study design and participants

This research is a cross-sectional analysis within the frame of the PA, Sedentarism and Obesity in Spanish Youth (PASOS) study, an observational, nationally representative, and multicenter study. Details of the PASOS study protocol has been fully described [24].

Eligible participants were children and adolescents aged 8–16 years who were enrolled in a participating school. Individuals with an intellectual disability that prevents response to the lifestyle questionnaires were excluded of the study. Each case was evaluated with the corresponding teachers and parents or legal guardians before exclusion. The PASOS study recruited a representative random sample of Spanish children and adolescents [24]. The initial sample was composed of 4092 participants (aged 8–16 years old) from March 2019 to February 2020 in 245 primary and secondary schools in 121 localities from each of the 17 Spanish autonomous communities (Ceuta and Melilla, two autonomous cities in North Africa with less than 0.8% of the total Spanish population aged 8–16 years, were not included for logistical reasons).

Participants were classified into two groups according to their type of municipality (i.e., rural or urban). Municipalities were categorized as rural areas if they had < 20,000 residents, and urban areas if they had ≥ 20,000 residents [8, 25, 26]. The participants who had not completed data on their age, type of area and school postal address were excluded from the study (n = 277). Therefore, 3815 participants (51.3% female) were included in the final sample, with a mean age of 12.5 years (SD ± 2.4). The distribution of the sample by type of municipality and age was: rural children (n = 581), urban children (n = 1145), rural adolescents (n = 659), and urban adolescents (n = 1430).

All parents and legal tutors of participants provided written informed consent. The study protocol and procedures were approved by the Ethics Committee of the Fundació Sant Joan de Déu, Barcelona, Spain. The trial was registered in 2019 at the International Standard Randomized Controlled Trial (ISRCT; https://doi.org/10.1186/ISRCTN34251612) with the number 34251612.

### Measures

#### Neighborhood walkability and SES measures

Walk Score was used to evaluate walkability of the neighborhood. According to previous research, Walk Score is a reliable and valid measure of estimating access to walkable amenities and PA behaviors[14, 27, 28]. The Walk Score has shown good validity in rural and urban settings [29]. Recent studies [15–17] suggest the validity of the Walk Score in the prediction of different types of pf physical activities measured in adult populations. The Walk Score algorithm produces a score range from 0 (lowest walkability) to 100 (highest walkability) for each postal address [29–31]. The methodology used by Walk Score is based on the shortest network distance to diverse amenities (e.g., shops, restaurants, schools, parks, recreation or entertainment facilities) within 1.5 miles and adjusted for street network attributes (e.g., intersection density and block length). The walkability score of each school attended by the participants was retrieved entering the postal address into the Walk Score website (www.walkscore.com). Moreover, as in previous research [23], median household income was used as an indicator of SES. Data on the household income of the each school census blocks were obtained through the National Institute of Statistics for 2017. According to the current literature (e.g., Molina-García et al., 2020 [8]), Spanish children and adolescents live less than 600 meters from the school. Hence, the present study has analyzed the walkability and SES characteristics of the school neighborhoods as representative of the neighborhoods in which participants live.

Walkability and SES values for our study were normalized using z-score transformation. As in previous studies (e.g., Frank et al., 2010 [11]; Molina-García et al., 2017 [12]), walkability and SES values were divided into deciles: the highest five deciles constituted the “high” category, and the lowest five deciles corresponded with “low” category. A 2x2 matrix was stablished by high/low walkability and high/low SES, with the four categories called "quadrants". The use of binary variables (e.g., high or low) allows comparison with other previous research from diverse geographical contexts [23].

#### Physical activity

A 7-item self-reported validated questionnaire, was used to assess PA levels in each participating child or adolescent [32]. Six questions ask about PA frequency and duration in the previous week: (1) How many days did you go for a walk? (2) How many days did you participate in movement play during recess time? (3) How many days did you participate in movement play during free time after school or during the weekend? (4) How many days did you have PE class at school? (5) How many days did you play a team sport? (6) How many days did you play an individual sport? The response options for these questions about PA were presented in a table with a box for each day of the week, in which children could mark if they have spent: (1) 0 min (no activity); (2) less than 30 min; (3) between 30 min and 1 hour; (4) between 1 hour and 1.5 hours; or (5) more than 1.5 hours. The continuous variable was created using the mean score for each category and after adding them all up (i.e. 1 = 0; 2 = 15min; 3 = 45 min, 4 = 75 min and 5 = 100 min). In the present study, three types of variables were created for the different PA behaviours (i.e., walk, play, sports team and individual sport): “per day”, average PA considering all days of the week; “per weekday”, average PA considering only the days from Monday to Friday; and “per weekend day”, average PA taking into account Saturday and Sunday. Moreover, the number of days (per week) physically active ≥ 60 min/day of MVPA was used in the present study.

In addition, in a subsample of 10% of the participants, randomly selected from the entire sample, PA was objectively measured by accelerometers for 9 days, these children and adolescents wore the ActiGraph wGT3X-BT (Pensacola, FL, USA) accelerometer. The random selection of the subsample was based on the use of a computerized random number generation process. Total PA, PA intensity, sedentary time and sleep duration were recorded. In the present study, total PA during both weekdays and weekend day were used. Furthermore, three types of variables were created for MVPA (i.e, per day, per weekday and, per weekend day).

### Data analyses

Descriptive statistics (e.g., means, standard deviations, skewness for continuous measures, frequencies, and percentages) were computed to analyze the distributions of the measurements.

The main variables of interest in the study models were high versus low neighborhood walkability and high versus low neighborhood SES, as well as the interaction between these two factors. For each PA outcome, the full model (walkability and SES main effects, interactions, and covariates) was first tested to determine whether there was an SES -walkability interaction-effect. Separate mixed-effect regression models (using SPSS MIXED) were fit for all the dependent variables (i.e., PA outcomes). Considering previous research [12, 33], mixed-regression analyses were used so that clustering of participants nested within school neighborhoods (administrative units) and municipality could be adjusted for as random effects. First, models were performed for the whole sample, and then separately for rural children, urban children, rural adolescents, and urban adolescents. Models were computed controlling for participant’s gender, age and family educational level. In relation to family educational level, participants’ parents responded between 1 (“No education”) to 6 (“University degree”). All analyses were carried out using SPSS 26.

## Results

Table 1 shows the study descriptive characteristics for all participants and differentiating by type of municipality and age groups. Valid accelerometer data were available for 9.4% of participants (i.e., 359 adolescents).

**Table 1:** Descriptive characteristics of the sample by residential area and age group.

	All	Rural Children	Urban Children	Rural Adolescents	Urban Adolescents
*Socio-demographics*					
Gender (n (%))					
Male	1855 (48.6)	290 (49.9)	573 (50.0)	312 (47.3)	680 (47.6)
Female	1959 (51.3)	291 (50.1)	572 (50.0)	347 (52.7)	749 (52.4)
Age (years)	12.5 (2.4)	10.4 (1.0)	10.3 (1.1)	14.4 (1.4)	14.3 (1.4)
Family educational level	2.7 (1.4)	2.7 (1.4)	2.6 (1.4)	2.8 (1.5)	2.6 (1.4)
*Physical activity outcomes*					
Walk (min/day)	50.2 (25.9)	47.2 (26.9)	46.6 (24.7)	53.0 (24.8)	53.1 (26.5)
Walk (min/weekday)	49.1 (27.7)	45.9 (28.7)	44.9 (26.7)	52.1 (26.6)	52.4 (27.9)
Walk (min/weekend day)	52.9 (33.4)	50.4 (33.9)	50.7 (32.5)	55.4 (32.5)	54.6 (34.1)
Play (min/day)	43.7 (28.1)	47.6 (27.3)	46.3 (26.7)	41.6 (28.8)	41.2 (28.9)
Play (min/weekday)	42.5 (29.5)	46.2 (28.4)	44.6 (28.5)	41.0 (30.1)	40.2 (30.2)
Play (min/weekend day)	46.8 (34.8)	51.1 (34.9)	50.6 (34.0)	43.2 (34.7)	43.7 (35.1)
Sports team (min/day)	31.5 (28.0)	34.0 (26.4)	33.5 (26.7)	28.9 (28.9)	30.3 (28.9)
Sports team (min/weekday)	32.4 (28.9)	34.3 (27.0)	34.3 (27.5)	29.8 (29.8)	31.3 (30.2)
Sports team (min/weekend day)	29.3 (33.8)	33.1 (34.5)	31.3 (33.4)	26.6 (33.4)	27.6 (33.8)
Individual sport (min/day)	25.4 (26.8)	28.2 (26.6)	27.1 (25.8)	22.7 (26.9)	24.3 (27.4)
Individual sport (min/weekday)	26.5 (28.1)	29.0 (27.1)	27.9 (26.9)	23.4 (28.2)	25.9 (29.2)
Individual sport (min/weekend day)	22.7 (31.3)	26.2 (32.6)	25.1 (31.7)	20.8 (30.6)	20.2 (30.4)
Physically active ≥ 60 min/day of MVPA (days per week)	4.9 (2.2)	5.6 (1.9)	5.5 (1.9)	4.4 (2.4)	4.4 (2.4)
MVPA (min/day)^a^	93.5 (35.1)	118.7 (24.5)	109.6 (30.3)	70.6 (29.3)	76.8 (31.1)
MVPA (min/weekday)	98.0 (36.7)	122.1 (27.2)	116.5 (30.6)	74.1 (28.1)	80.1 (33.2)
MVPA (min/weekend day)	74.4 (42.8)	103.1 (41.9)	83.0 (43.1)	50.3 (39.5)	62.6 (35.2)

Note. MVPA: moderate to vigorous physical activity. Data from a subsample of 359 participants: rural children (n = 60), urban children (n = 114), rural adolescents (n = 42), and urban adolescents (n = 143).

PA outcomes with covariate-adjusted means by neighborhood quadrants are shown in Table 2 for the whole sample. The SES-walkability interaction or main effects of SES and walkability is also shown. According to Table 2, a significant SES-walkability interaction was found for walking behavior on weekend days (p = 0.023). The lowest average minutes spent in walking behavior was found in neighborhoods classified as low-SES/low-walkable. Participants from this kind of quadrant accumulated almost 5 less minutes per day walking than participants from the other three types of neighborhoods. Moreover, there was one main effect of neighborhood walkability on walking behavior (p = 0.042). Youth from more walkable areas reported more minutes per day compared with those from less walkable neighborhoods (51.4 vs 48.8 minutes, respectively).

**Table 2:** Mixed effects regression models, for the whole sample, between neighborhood socioeconomic status (SES)-by-walkability interaction, and the main effects of walkability and SES without interaction.

	Low walkability		High walkability		SES-by-walkability interaction (*p* value)			Walkability main effect (*p* value)			SES main effect (*p* value)
	SES		SES			Walkability			SES		
Outcome variables	Low	High	Low	High		Low	High		Low	High	
Walk (min/day)	48.5 (1.1)	49.2 (1.4)	52.3 (1.4)	50.6 (1.2)	0.323	48.8 (0.9)	51.4 (1.0)	**0.042**	50.0 (0.9)	49.9 (0.9)	0.989
Walk (min/weekday)	48.2 (1.2)	47.4 (1.5)	51.1 (1.5)	49.5 (1.3)	0.747	47.9 (0.9)	50.1 (1.0)	0.092	49.3 (1.0)	48.5 (1.0)	0.571
Walk (min/weekend day)	49.4 (1.3)	54.0 (1.6)	55.2 (1.6)	53.3 (1.4)	**0.023**	51.3 (1.1)	54.0 (1.1)	0.061	51.7 (1.1)	53.5 (1.1)	0.208
Play (min/day)	42.8 (1.1)	45.9 (1.3)	44.1 (1.4)	42.6 (1.2)	0.060	44.1 (0.8)	43.2 (0.9)	0.466	43.3 (0.9)	44.0 (0.9)	0.563
Play (min/weekday)	42.2 (1.1)	44.3 (1.3)	42.7 (1.4)	41.1 (1.2)	0.126	43.1 (0.8)	41.8 (0.9)	0.289	42.4 (0.9)	42.5 (0.9)	0.950
Play (min/weekend day)	44.3 (1.4)	49.9 (1.6)	47.7 (1.7)	46.0 (1.5)	**0.015**	46.6 (1.1)	46.6 (1.2)	0.982	45.6 (1.1)	47.7 (1.1)	0.184
Sports team (min/day)	29.8 (1.2)	32.4 (1.4)	31.1 (1.5)	32.7 (1.3)	0.680	30.8 (0.9)	32.0 (1.0)	0.377	30.3 (0.9)	32.5 (1.0)	0.092
Sports team (min/weekday)	31.3 (1.2)	32.6 (1.5)	32.0 (1.5)	33.4 (1.3)	0.979	31.8 (0.9)	32.8 (1.0)	0.474	31.5 (0.9)	33.0 (1.0)	0.262
Sports team (min/weekend day)	26.1 (1.4)	31.6 (1.7)	29.3 (1.7)	30.9 (1.5)	0.194	28.3 (1.1)	30.2 (1.2)	0.247	27.4 (1.1)	31.1 (1.2)	**0.017**
Individual sport (min/day)	23.7 (1.1)	26.6 (1.4)	24.5 (1.4)	25.7 (1.2)	0.509	24.9 (0.9)	25.1 (0.9)	0.839	24.0 (0.9)	26.1 (0.9)	0.098
Individual sport (min/weekday)	24.9 (1.2)	27.6 (1.4)	25.5 (1.5)	26.9 (1.3)	0.610	26.0 (0.9)	26.3 (1.0)	0.834	25.2 (0.9)	27.2 (1.0)	0.124
Individual sport (min/weekend day)	20.7 (1.2)	24.1 (1.5)	21.8 (1.5)	22.8 (1.3)	0.383	22.1 (1.0)	22.3 (1.0)	0.892	21.1 (1.0)	23.3 (1.0)	0.115
Physically active ≥ 60 min/day of MVPA (days per week)	4.6 (0.1)	5.1 (0.1)	4.9 (0.1)	5.0 (0.1)	0.096	4.8 (0.1)	5.0 (0.1)	0.305	4.7 (0.1)	5.1 (0.1)	**0.002**
MVPA (min/day)	92.6 (7.7)	81.3 (6.0)	95.9 (5.1)	93.5 (4.5)	0.478	85.6 (4.9)	94.6 (3.5)	0.160	95.3 (4.6)	89.0 (4.0)	0.324
MVPA (min/weekday)	100.4 (8.7)	84.6 (6.9)	102.6 (5.7)	97.6 (5.2)	0.462	90.8 (5.8)	100.0 (4.1)	0.222	102.3 (5.2)	92.8 (4.5)	0.195
MVPA (min/weekend day)	70.3 (10.1)	61.8 (7.9)	74.1 (6.7)	76.2 (6.0)	0.524	65.1 (6.2)	75.3 (4.4)	0.203	73.2 (5.9)	70.9 (5.0)	0.766

Note. MVPA: moderate to vigorous physical activity. Bold values indicate statistically significant differences (*p* < 0.05). All models included the following covariates: participant’s gender, age and family educational level. Adjusted means (SD) are presented in the table.

There was another SES-walkability interaction for the play behavior involving weekend days (p = 0.015) (Table 2). The lowest average minutes spent in playing (44.3 minutes/day) was found among participants from low-SES and low-walkable neighborhoods, in contrast to the other three quadrants (average of 47.9 minutes). Also, the participation in sports team during weekend and the number of days being active ≥ 60 minutes/day showed significant SES main effects (p = 0.017 and p = 0.002, respectively). The values of the both variables were higher in high-SES neighborhoods.

For rural children (Table 3), there was one neighborhood walkability main effect, with children from higher-walkability neighborhoods reporting a greater participation in sports team, compared to their peers from lower-walkability neighborhoods (p < 0.05). In the case of urban children (Table 4), there was one significant SES-walkability interaction for play behavior (p = 0.020). Play behavior was less frequent during weekend days in areas classified as low-SES/low-walkable. Urban children from this kind of quadrant accumulated almost 9 less minutes per day of playing time than children from the other three types of neighborhoods. Moreover, the participation in sports team and the number of days being active ≥ 60 minutes/day were more frequent in areas with high SES among urban children (p < 0.05). Finally, there was another main effect of neighborhood walkability on MVPA measured by accelerometry, both on weekdays and weekend days (p = 0.048 and p = 0.000, respectively). Urban children from more walkable areas accumulated more minutes per day compared with those from less walkable neighborhoods. This difference was almost 50 minutes during the weekend days (103.3 vs 53.6 minutes).

**Table 3:** Mixed effects regression models, for rural children, between neighborhood socioeconomic status (SES)-by-walkability interaction, and the main effects of walkability and SES without interaction.

	Low walkability		High walkability		SES-by-walkability interaction (*p* value)			Walkability main effect (*p* value)			SES main effect (*p* value)
	SES		SES			Walkability			SES		
Outcome variables	Low	High	Low	High		Low	High		Low	High	
Walk (min/day)	44.8 (2.5)	46.6 (3.4)	54.2 (5.4)	52.7 (12.9)	0.823	45.5 (2.0)	53.9 (5.1)	0.131	46.5 (2.4)	47.0 (3.4)	0.911
Walk (min/weekday)	43.4 (2.8)	45.0 (3.7)	52.2 (5.9)	52.3 (14.0)	0.927	44.0 (2.2)	52.2 (5.5)	0.177	45.0 (2.5)	45.5 (3.6)	0.921
Walk (min/weekend day)	48.1 (2.7)	50.1 (3.4)	58.7 (5.5)	53.3 (14.5)	0.642	48.9 (2.1)	58.0 (5.2)	0.117	50.2 (2.5)	50.3 (3.5)	0.965
Play (min/day)	46.9 (1.7)	46.6 (2.0)	47.0 (3.2)	54.1 (10.1)	0.489	46.8 (1.3)	47.7 (3.1)	0.790	47.0 (1.5)	46.8 (1.9)	0.948
Play (min/weekday)	45.8 (1.8)	45.1 (2.1)	44.7 (3.4)	53.1 (10.5)	0.419	45.5 (1.3)	45.5 (3.2)	0.998	45.6 (1.6)	45.4 (2.0)	0.932
Play (min/weekend day)	50.4 (2.8)	51.1 (3.6)	53.9 (5.8)	57.2 (15.2)	0.881	50.6 (2.2)	54.3 (5.4)	0.536	51.1 (2.5)	51.4 (3.5)	0.939
Sports team (min/day)	31.3 (1.6)	33.8 (1.9)	38.9 (3.1)	45.9 (9.5)	0.660	32.4 (1.2)	39.5 (2.9)	**0.025**	33.0 (1.4)	34.2 (1.9)	0.618
Sports team (min/weekday)	32.2 (1.6)	34.4 (1.9)	37.8 (3.1)	46.5 (9.8)	0.536	33.1 (1.2)	38.6 (3.0)	0.098	33.5 (1.5)	34.8 (1.9)	0.591
Sports team (min/weekend day)	29.2 (2.1)	32.5 (2.5)	41.8 (4.1)	44.6 (12.7)	0.969	30.6 (1.6)	42.0 (3.9)	**0.007**	31.9 (1.9)	32.8 (2.5)	0.783
Individual sport (min/day)	25.5 (1.6)	27.2 (1.9)	31.3 (3.1)	20.9 (9.7)	0.239	26.2 (1.2)	30.4 (3.0)	0.195	26.7 (1.4)	27.0 (1.9)	0.909
Individual sport (min/weekday)	25.7 (1.6)	28.9 (1.9)	31.3 (3.1)	20.0 (9.8)	0.171	26.7 (1.9)	29.0 (4.7)	0.657	26.8 (2.2)	27.4 (3.1)	0.868
Individual sport (min/weekend day)	24.8 (2.0)	23.2 (2.4)	31.5 (3.9)	23.3 (12.1)	0.606	24.1 (1.5)	30.8 (3.7)	0.096	26.2 (1.8)	23.2 (2.3)	0.308
Physically active ≥ 60 min/day of MVPA (days per week)	5.3 (0.2)	5.8 (0.2)	6.1 (0.3)	5.5 (0.8)	0.255	5.5 (0.1)	6.0 (0.3)	0.173	5.5 (0.2)	5.8 (0.2)	0.289
MVPA (min/day)^a^											
MVPA (min/weekday)											
MVPA (min/weekend day)											

Note. MVPA: moderate to vigorous physical activity. Bold values indicate statistically significant differences (*p* < 0.05). All models included the following covariates: participant’s gender, age and family educational level. Adjusted means (SD) are presented in the table. ^a^Insufficient number of participants to fit any models.

**Table 4:** Mixed effects regression models, for urban children, between neighborhood socioeconomic status (SES)-by-walkability interaction, and the main effects of walkability and SES without interaction.

	Low walkability		High walkability		SES-by-walkability interaction (*p* value)			Walkability main effect (*p* value)			SES main effect (*p* value)
	SES		SES			Walkability			SES		
Outcome variables	Low	High	Low	High		Low	High		Low	High	
Walk (min/day)	44.2 (2.0)	46.3 (2.5)	47.0 (1.9)	46.7 (1.5)	0.549	45.0 (1.6)	46.9 (1.2)	0.361	45.8 (1.4)	46.6 (1.3)	0.653
Walk (min/weekday)	43.6 (2.1)	44.2 (2.6)	45.0 (2.0)	45.1 (1.6)	0.904	43.8 (1.7)	45.0 (1.2)	0.560	44.3 (1.4)	44.8 (1.4)	0.801
Walk (min/weekend day)	46.0 (2.6)	51.6 (3.3)	52.3 (2.4)	51.0 (2.0)	0.185	48.1 (2.1)	51.5 (1.6)	0.201	49.3 (1.8)	51.1 (1.7)	0.476
Play (min/day)	43.6 (2.2)	51.1 (2.7)	46.1 (2.0)	45.9 (1.6)	0.078	46.4 (1.8)	46.0 (1.3)	0.836	44.9 (1.6)	47.3 (1.5)	0.266
Play (min/weekday)	43.6 (2.3)	49.1 (2.9)	43.7 (2.1)	43.9 (1.7)	0.233	45.7 (1.8)	43.8 (1.4)	0.409	43.6 (1.6)	45.3 (1.5)	0.445
Play (min/weekend day)	44.1 (2.9)	56.1 (3.6)	52.1 (2.7)	50.9 (2.2)	**0.020**	48.6 (2.4)	51.3 (1.8)	0.367	48.2 (2.1)	52.2 (2.0)	0.159
Sports team (min/day)	31.4 (2.7)	37.5 (3.4)	28.2 (2.5)	35.3 (2.1)	0.861	33.9 (2.2)	32.2 (1.7)	0.541	29.6 (1.9)	35.9 (1.8)	**0.019**
Sports team (min/weekday)	33.7 (2.6)	38.8 (3.4)	29.0 (2.5)	35.9 (2.1)	0.737	35.7 (2.1)	33.0 (2.6)	0.315	31.1 (1.8)	36.7 (1.8)	**0.032**
Sports team (min/weekend day)	25.9 (3.2)	34.1 (4.1)	26.6 (3.0)	34.1 (2.5)	0.920	29.0 (2.7)	30.9 (2.1)	0.573	26.3 (2.2)	34.1 (2.2)	**0.011**
Individual sport (min/day)	25.8 (2.4)	29.5 (3.0)	23.5 (2.3)	28.7 (1.9)	0.771	27.2 (1.9)	26.6 (1.5)	0.805	24.6 (1.7)	28.9 (1.6)	0.059
Individual sport (min/weekday)	27.4 (2.5)	30.3 (3.1)	23.9 (2.3)	29.5 (1.9)	0.579	28.5 (2.0)	27.3 (1.5)	0.613	25.5 (1.7)	29.7 (1.7)	0.077
Individual sport (min/weekend day)	21.9 (2.9)	27.9 (3.6)	22.6 (2.7)	26.4 (2.2)	0.712	24.2 (2.3)	24.9 (1.8)	0.813	22.3 (2.0)	26.8 (1.9)	0.100
Physically active ≥ 60 min/day of MVPA (days per week)	5.0 (0.2)	5.8 (0.2)	5.2 (0.2)	5.7 (0.1)	0.367	5.3 (0.1)	5.5 (0.1)	0.295	5.1 (0.1)	5.7 (0.1)	**0.001**
MVPA (min/day)	116.4 (15.0)	87.3 (11.0)	124.5 (5.5)	120.3 (5.2)	0.288	98.8 (7.3)	121.7 (3.8)	**0.008**	122.5 (8.0)	109.7 (7.0)	0.282
MVPA (min/weekday)	133.6 (15.8)	94.9 (11.6)	131.4 (5.8)	126.3 (5.5)	0.175	110.2 (7.8)	128.0 (4.0)	**0.048**	131.5 (8.0)	116.0 (7.0)	0.202
MVPA (min/weekend day)	67.9 (19.9)	44.4 (14.6)	104.6 (7.2)	103.0 (6.9)	0.480	53.6 (9.6)	103.3 (4.9)	**0.000**	95.1 (14.6)	84.6 (13.1)	0.616

Note. MVPA: moderate to vigorous physical activity. Bold values indicate statistically significant differences (*p* < 0.05). All models included the following covariates: participant’s gender, age and family educational level. Adjusted means (SD) are presented in the table.

In relation to rural adolescents (Table 5), the participation in sports team and individual sports during weekend days, was more frequent overall in higher-SES areas (p < 0.05). Also, the number of days being active ≥ 60 minutes/day was higher in areas with high SES (p < 0.05). Finally, regarding urban adolescents (Table 6), there was a SES-walkability interaction for the walking behavior on weekend days (p = 0.025). The lowest average minutes spent in walking (48.4 minutes/day) was found among participants from low-SES and low-walkable neighborhoods, in contrast to the other three quadrants (average of 56.0 minutes).

**Table 5:** Mixed effects regression models, for rural adolescents, between neighborhood socioeconomic status (SES)-by-walkability interaction, and the main effects of walkability and SES without interaction.

	Low walkability		High walkability		SES-by-walkability interaction (*p* value)			Walkability main effect (*p* value)			SES main effect (*p* value)
	SES		SES			Walkability			SES		
Outcome variables	Low	High	Low	High		Low	High		Low	High	
Walk (min/day)	53.3 (1.6)	50.7 (2.3)	53.1 (6.7)	60.2 (5.2)	0.2.84	52.4 (1.3)	57.7 (4.2)	0.242	53.3 (1.6)	52.2 (2.1)	0.677
Walk (min/weekday)	53.2 (1.8)	47.8 (2.4)	56.2 (7.3)	60.8 (5.6)	0.313	51.3 (1.5)	59.4 (4.7)	0.110	53.4 (1.8)	49.9 (2.3)	0.248
Walk (min/weekend day)	53.9 (1.8)	58.3 (2.6)	45.8 (6.9)	58.0 (6.1)	0.425	55.3 (1.5)	52.6 (4.8)	0.604	53.3 (1.8)	58.3 (2.4)	0.112
Play (min/day)	40.2 (1.4)	43.2 (2.1)	40.1 (5.6)	49.8 (5.0)	0.398	41.2 (1.2)	45.3 (3.8)	0.302	40.2 (1.4)	44.2 (1.9)	0.104
Play (min/weekday)	39.8 (1.5)	41.5 (2.2)	41.3 (6.0)	51.9 (5.3)	0.294	40.4 (1.2)	47.1 (4.0)	0.110	39.9 (1.5)	43.0 (2.1)	0.227
Play (min/weekend day)	41.5 (2.1)	47.8 (3.0)	38.0 (8.0)	44.4 (6.7)	0.996	43.6 (1.8)	41.6 (5.3)	0.722	41.3 (2.0)	47.3 (2.8)	0.092
Sports team (min/day)	27.1 (1.5)	29.7 (2.1)	33.4 (5.8)	36.9 (5.1)	0.907	28.0 (1.2)	35.2 (3.9)	0.083	27.6 (1.5)	30.7 (2.0)	0.224
Sports team (min/weekday)	28.9 (1.6)	29.1 (2.2)	34.7 (6.1)	37.0 (5.3)	0.805	29.0 (1.3)	35.9 (4.0)	0.109	29.3 (1.5)	30.2 (2.1)	0.750
Sports team (min/weekend day)	22.6 (1.9)	31.3 (2.7)	29.9 (7.2)	36.6 (6.2)	0.846	25.5 (1.7)	33.4 (5.0)	0.142	23.1 (1.8)	32.1 (2.5)	**0.007**
Individual sport (min/day)	20.1 (1.5)	23.5 (2.2)	21.2 (5.8)	30.2 (5.0)	0.484	21.2 (1.3)	26.3 (3.9)	0.220	20.2 (1.5)	24.5 (2.0)	0.096
Individual sport (min/weekday)	21.3 (1.6)	23.0 (2.3)	21.6 (6.2)	33.1 (5.3)	0.263	21.9 (1.4)	28.2 (4.1)	0.151	21.3 (1.6)	24.6 (2.2)	0.235
Individual sport (min/weekend day)	17.2 (1.5)	24.5 (2.2)	20.0 (6.0)	23.3 (5.4)	0.648	19.6 (1.3)	21.5 (4.1)	0.659	17.4 (1.5)	24.3 (2.1)	**0.008**
Physically active ≥ 60 min/day of MVPA (days per week)	4.2 (0.1)	4.7 (0.2)	4.6 (0.5)	5.0 (0.4)	0.836	4.4 (0.1)	4.8 (0.3)	0.225	4.2 (0.1)	4.7 (0.2)	**0.025**
MVPA (min/day)^a^											
MVPA (min/weekday)											
MVPA (min/weekend day)											

Note. MVPA: moderate to vigorous physical activity. Bold values indicate statistically significant differences (*p* < 0.05). All models included the following covariates: participant’s gender, age and family educational level. Adjusted means (SD) are presented in the table. ^a^Insufficient number of participants to fit any models.

**Table 6:** Mixed effects regression models, for urban adolescents, between neighborhood socioeconomic status (SES)-by-walkability interaction, and the main effects of walkability and SES without interaction.

	Low walkability		High walkability		SES-by-walkability interaction (*p* value)			Walkability main effect (*p* value)			SES main effect (*p* value)
	SES		SES			Walkability			SES		
Outcome variables	Low	High	Low	High		Low	High		Low	High	
Walk (min/day)	50.2 (2.3)	53.3 (2.2)	55.4 (1.9)	52.8 (1.5)	0.157	51.8 (1.6)	53.8 (1.2)	0.306	53.3 (1.5)	52.9 (1.2)	0.854
Walk (min/weekday)	50.8 (2.5)	51.9 (2.4)	54.8 (2.0)	52.1 (1.6)	0.386	51.4 (1.7)	53.1 (1.3)	0.410	53.2 (1.6)	52.0 (1.3)	0.587
Walk (min/weekend day)	48.4 (2.7)	56.8 (2.6)	56.8 (2.2)	54.4 (1.7)	**0.025**	52.8 (1.9)	55.3 (1.4)	0.286	53.5 (1.8)	55.1 (1.5)	0.481
Play (min/day)	41.3 (2.2)	43.9 (2.1)	41.8 (1.8)	39.7 (1.4)	0.228	42.6 (1.5)	40.5 (1.1)	0.266	41.6 (1.4)	41.0 (1.2)	0.726
Play (min/weekday)	40.1 (2.5)	42.6 (2.4)	41.5 (2.0)	38.5 (1.6)	0.193	41.4 (1.7)	39.6 (1.2)	0.400	40.9 (1.6)	39.7 (1.3)	0.564
Play (min/weekend day)	43.9 (2.5)	47.2 (2.3)	42.9 (2.0)	42.7 (1.6)	0.414	45.7 (1.7)	42.7 (1.2)	0.169	43.3 (1.6)	44.1 (1.3)	0.720
Sports team (min/day)	30.6 (2.4)	30.4 (2.3)	31.5 (1.9)	29.9 (1.5)	0.729	30.5 (1.7)	30.5 (1.2)	0.989	31.2 (1.5)	30.1 (1.3)	0.584
Sports team (min/weekday)	31.8 (2.5)	31.0 (2.4)	32.9 (2.0)	31.0 (1.6)	0.790	31.4 (1.7)	31.7 (1.2)	0.887	32.4 (1.6)	31.0 (1.3)	0.484
Sports team (min/weekend day)	27.9 (2.8)	28.7 (2.7)	28.1 (2.3)	27.3 (1.8)	0.748	28.3 (1.9)	27.6 (1.4)	0.758	28.1 (1.8)	27.7 (1.5)	0.870
Individual sport (min/day)	24.9 (2.3)	25.7 (2.2)	24.3 (1.8)	23.2 (1.5)	0.619	25.4 (1.6)	23.6 (1.2)	0.351	24.6 (1.4)	23.9 (1.2)	0.740
Individual sport (min/weekday)	27.0 (2.4)	27.3 (2.3)	25.9 (2.0)	24.6 (1.6)	0.698	27.2 (1.7)	25.1 (1.2)	0.308	26.4 (1.5)	25.5 (1.3)	0.644
Individual sport (min/weekend day)	20.1 (2.5)	21.6 (2.4)	19.8 (2.0)	19.3 (1.6)	0.653	20.9 (1.7)	19.5 (1.3)	0.499	19.9 (1.6)	20.0 (1.4)	0.972
Physically active ≥ 60 min/day of MVPA (days per week)	4.4 (0.2)	4.6 (0.2)	4.4 (0.2)	4.5 (0.1)	0.693	4.5 (0.1)	4.4 (0.1)	0.862	4.4 (0.1)	4.5 (0.1)	0.439
MVPA (min/day)	76.5 (6.2)	64.8 (6.2)	87.0 (5.8)	77.2 (3.5)	0.855	72.1 (5.3)	80.2 (3.6)	0.264	83.1 (5.3)	74.5 (4.3)	0.249
MVPA (min/weekday)	82.1 (7.7)	67.3 (8.5)	92.2 (7.2)	80.5 (4.7)	0.835	77.4 (7.2)	84.4 (4.9)	0.452	88.4 (6.4)	77.9 (5.3)	0.252
MVPA (min/weekend day)	66.6 (7.5)	57.4 (7.5)	69.2 (7.1)	63.1 (4.3)	0.821	62.1 (5.3)	64.8 (3.7)	0.681	68.0 (5.1)	61.7 (3.8)	0.328

Note. MVPA: moderate to vigorous physical activity. Bold values indicate statistically significant differences (*p* < 0.05). All models included the following covariates: participant’s gender, age and family educational level. Adjusted means (SD) are presented in the table.

## Discussion

This national representative study highlights the importance of neighborhood’s walkability and SES when designing interventions to promote PA behavior. This study is in line with previous research in adolescents [12, 18] and extends the current knowledge by reporting significant interactions between walkability and SES in relation to specific physical activities among a representative sample of Spanish youth.

One of the most relevant findings showed that for Spanish children and adolescents there was one main effect of neighborhood walkability on all week walking behavior independently from SES. Considering that walking is the easiest type of PA and one of the greatest opportunities to increase PA levels, our results are of great importance because they bring an opportunity to promote PA, even in low SES neighborhoods. Built environment, in particular neighborhood walkability has also been shown to be especially important to promote PA for adolescents with perceived low self-efficacy to be active and low levels of MVPA [34].

Moreover, a significant SES-walkability interaction was found during weekend days for walking behavior (p = 0.023) and play behavior (p = 0.015). Weekend days offer more free time opportunities than during week days, but it seems that time spent playing outdoors and walking is lower at neighborhoods classified as low-SES/low-walkable versus high-SES/high-walkable (44.3 vs 46 min and 49.4 vs 53.3 min respectively). These results mean that during children’s free time, spontaneous forms of PA behavior, such as walking and playing outdoors, seem to be dependent on high SES and high neighborhood walkability.

SES neighborhoods influenced on the participation in team sports during the weekend, being this participation higher in high SES neighborhoods. Thus, organized sports participation was dependent mainly on SES and not on neighborhood’s walkability. These results are in line with Molina-García et al. [12] who suggested that the number of sports facilities and organized physical activities are normally lower in low-SES neighborhoods. Also, in relation to the achievement of children’s PA guidelines, children from high-SES neighborhoods accounted for a higher number of days being active ≥ 60 minutes/day (5.1 vs 4.7 days/week). According to our results, the socio-economic context in which young Spanish people live would directly affect their opportunities to have a physically active lifestyle. The most disadvantaged children and adolescents are less likely to engage in regular PA. Our findings are also in line with previous studies in adults, for instance, Cereijo et al. [35] who analyzed the relationship between area-level SES and availability of exercise facilities in Madrid, Spain. In this study, the overall number of facilities was lower in low SES areas compared with higher SES areas. The authors suggested that a possible intervention to improve health equity may be to increase the number of facilities in low SES environments.

When analyzing the results for children regarding rural versus urban environments, different main effects were found. It seems that neighborhood walkability was more important for children in rural environments, where children from higher-walkability neighborhoods reported a greater participation in sports team than children from lower-walkability neighborhoods during all week and, in particular, during weekend days. However, for urban children, only SES-interaction was responsible for sports participation during all week, weekdays, and weekend days, where children from low-SES environments reported less sports team participation than the ones from high SES environments. The lack of sports facilities in low SES environments could also be a probable reason [12]. One interesting result is the fact that in rural areas walkability seems to be more important to promote sports participation in children regardless of SES, which highlights the need to make built environments more walkable in either rural or urban neighborhoods. Moreover, the number of days being active ≥ 60 minutes/day in urban children with high SES was significantly higher than in lower SES environments (5.7 vs 5.1 days/week).

When analyzing the results for rural adolescents, only neighborhood SES was responsible for a higher participation during weekends in individual or team sports where adolescents from high SES environments presented more time spent in sports participation. Walkability seems not to be relevant for adolescents’ sports participation. Moreover, a SES effect was also found for the number of days achieving PA guidelines, which was higher for adolescents from high SES environments versus those from low SES ones (4.7 vs 4.2 days/week). Urban adolescents however, showed only a SES-walkability interaction for walking behavior on weekend days, where adolescents from low-SES and low-walkable neighborhoods reported lower walking behavior than those from a high-SES and high-walkable neighborhoods (48.4 vs 54.4 min).

The strengths of the present study included the use of a representative sample of Spanish children and adolescents that allow a greater generalizability of the findings. Another strength was the use of both objective and subjective measures of PA. A limitation was the cross-sectional design of the study that did not allow to establish cause-effect relationships.

## Conclusions

Therefore, according to the present findings, policy-makers and practitioners, when designing future community-based interventions for promoting active lifestyles among Spanish children and adolescents should consider the following:

- Providing high walkable environments seems a good strategy to promote PA regardless SES levels. It seems that improving the walkability is a key component to partially overcome the SES inequalities, especially in urban areas with low SES.
- The important role of the influence of high walkable neighborhoods, particularly for children, and the interaction of SES-Walkability effect for all.
- High-SES environments can offer better sports facilities and more organized physical activities than low-SES ones.

This is the first nationally representative study which has analyzed the relationship between Walk Score, as a measured of walkability, and SES environments with different types of PA behaviors of Spanish children and adolescents. This research is a benchmark for the future design of planning policies and intervention studies aimed at increasing PA levels among Spanish children and adolescents, and potentially for other countries in Europe and beyond.
